# Modeling the Spread of Vector-Borne Diseases on Bipartite Networks

**DOI:** 10.1371/journal.pone.0013796

**Published:** 2010-11-12

**Authors:** Donal Bisanzio, Luigi Bertolotti, Laura Tomassone, Giusi Amore, Charlotte Ragagli, Alessandro Mannelli, Mario Giacobini, Paolo Provero

**Affiliations:** 1 Department of Animal Production, Epidemiology and Ecology, University of Torino, Torino, Italy; 2 Molecular Biotechnology Center, University of Torino, Torino, Italy; 3 Ministry of Forestry and Agricultural Politics, State Forestry Department, Territorial Unit for Biodiversity, Lucca, Italy; 4 Department of Genetics Biology and Biochemistry, University of Torino, Torino, Italy; Direccion General de Epidemiologia, Peru

## Abstract

**Background:**

Vector-borne diseases for which transmission occurs exclusively between vectors and hosts can be modeled as spreading on a bipartite network.

**Methodology/Principal Findings:**

In such models the spreading of the disease strongly depends on the degree distribution of the two classes of nodes. It is sufficient for one of the classes to have a scale-free degree distribution with a slow enough decay for the network to have asymptotically vanishing epidemic threshold. Data on the distribution of *Ixodes ricinus* ticks on mice and lizards from two independent studies are well described by a scale-free distribution compatible with an asymptotically vanishing epidemic threshold. The commonly used negative binomial, instead, cannot describe the right tail of the empirical distribution.

**Conclusions/Significance:**

The extreme aggregation of vectors on hosts, described by the power-law decay of the degree distribution, makes the epidemic threshold decrease with the size of the network and vanish asymptotically.

## Introduction

Many natural, social and technological systems can be described in terms of self-organized networks of interacting entities. These networks often display topological properties, such as scale-free degree distributions, community structure and small-world phenomenon, which set them apart from simpler networks such as lattices and random networks (see e.g. [1]).

In particular, the spreading of a transmissible disease can be studied by modeling the population as a network of individuals, in which an edge is placed between two individuals if there is the possibility of transmission between them. It was shown [2] that degree distribution of the network has dramatic consequences on the spreading of the disease: while regular lattices and random networks have a non-zero epidemic threshold, that is a critical value of transmission probability under which the disease eventually dies out, such threshold vanishes asymptotically in scale-free networks. The latter degree distribution is characteristic of many real-world networks, including social and computer networks on which human diseases and computer viruses propagate.

In some cases of practical interest modeling the propagation of the disease requires the introduction of a bipartite network [3], in which the nodes (individuals) belong to two mutually exclusive classes and the edges (transmission) can occur exclusively between individuals of different classes. For example, Gómez–Gardeñes et al. [4] studied the spreading of sexually transmitted diseases in heterosexual populations and showed that the bipartite nature of the network must be taken into account to model the behavior of the epidemic threshold.

In this work we introduce another context in which bipartite networks provide the natural framework to study epidemic spreading, namely those vector-borne diseases in which transmission occurs exclusively between vectors and hosts. The aim of this study is twofold: first, we show that the analysis of bipartite networks performed in [4] implies that the epidemic threshold vanishes asymptotically even for bipartite networks in which the degree distribution of one class of nodes is not scale-free, as long as the other class is scale-free with exponent 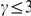.

Second, we analyze capture data of the tick *Ixodes ricinus* on their hosts. These ticks can transmit the pathogens responsible for Lyme disease (*Borrelia burgdorferi*) and the tick-borne encephalitis (TBE) virus (see [5], [6] for reviews). We show that the distribution of the number of ticks found on hosts indeed follows asymptotically a scale-free distribution compatible with a vanishing epidemic threshold.

## Results

### Epidemics spreading on bipartite networks

#### Analytical results

Consider a vector-borne disease which can only be transmitted between vectors and hosts, and can thus be modeled on a bipartite network. An edge placed between a host and a vector represents the possibility for the disease to spread from one to the other. Two separate degree distributions can be defined for hosts and vectors.

Gómez–Gardeñes and collaborators [4] recently studied disease spreading on scale-free bipartite graphs as a model of sexually transmitted diseases in heterosexual populations. In a mean-field approach they showed that the epidemic threshold for a bipartite network with nodes falling into classes 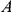 and 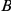 is given by
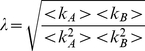
(1)


It follows that for the epidemic threshold to vanish asymptotically it is sufficient that the second moment of the degree distribution of *one* class of nodes diverges. Therefore it is enough for the hosts (or vectors) to have a scale-free distribution of contacts (with power-law exponent 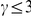) for the epidemic threshold to vanish in the limit of infinite network size.

#### Simulations

Since these results were obtained in a mean-field approximation, and the simulations corroborating them in [4] were performed in the case where both degree distributions are scale-free, we performed our own simulations in which the hosts have a scale-free degree distribution, but the number of edges touching a vector is Poisson-distributed. We simulated the SIS model on such networks, measuring the dependence of the epidemic threshold on the network size.

**Figure 1:**
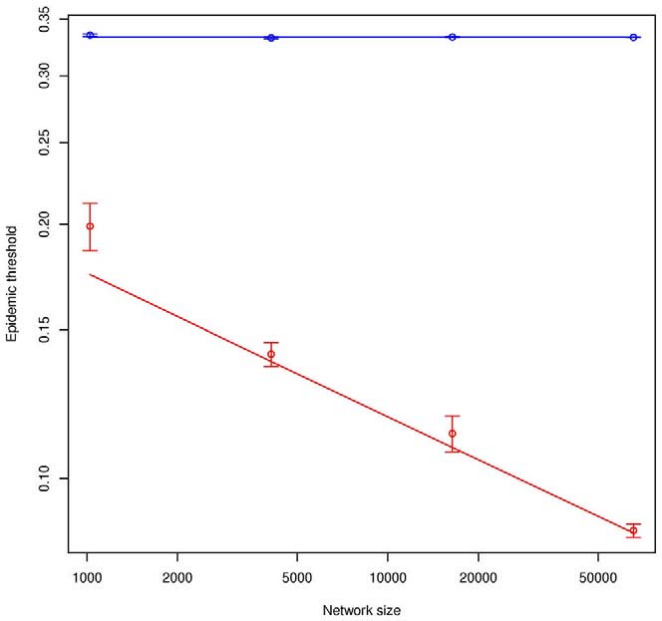
Dependence of the epidemic threshold on the network size for various degree distributions (logarithmic scale on both axes). The circles represent the epidemic threshold from our simulation, and the lines the predictions obtained with Eq.1. Colors refer to combinations of degree distributions. Blue: Poisson distribution for both hosts and vectors; red: Poisson distribution for vectors, scale-free (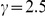) for hosts.

The results are shown in Fig. 1. When the degree distribution is scale-free for the host and Poisson for the vectors the epidemic threshold decreases with the network size following a power-law, in agreement with the predictions made using Eq.1. As expected, the epidemic threshold does not depend on the network size when both vectors and host follow the Poisson degree distribution. The conclusion is that the results of Ref. [4] hold also when one of the degree distributions is not scale-free.

As noted in [4] taking into account the bipartite nature of the network is necessary to get the correct scaling behavior of the epidemic threshold. Consider for example the case in which hosts have scale-free degree distribution while the one of the vectors is Poisson. If one were to study the non-bipartite network obtained by projecting on the hosts, one would get a scale-free network of hosts: the theory of epidemic propagation on such network would predict the epidemic threshold to behave as
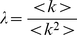
(2)where the expectation values are computed on the projected network (see [7] for a discussion of bipartite network projections). This in turn would lead to a scaling exponent equal to twice the one predicted for the bipartite network.

### A bipartite network model of Lyme disease and Tick-Borne Encephalitis

In this section we show that the results described above apply in particular to the propagation of two tick-borne pathogens, namely the Lyme disease agent *Borrelia burgdorferi* and the TBE virus, since:

1. Transmission between vectors requires a common host.
2. The degree distribution of the hosts is scale free, with exponent 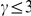.

It is well established that the main avenue for the transmission of these pathogens between vectors requires a host: the pathogen is either transmitted from a vector to a host and from the latter to another host, or from a vector to another one feeding nearby on the same host (co-feeding) [5], [6], [8]. Both modes of transmission require a host and thus create a bipartite network. The role of transovarial transmission, which is host-independent, is still under discussion, but the consensus seems to be that, by itself, this mode of transmission is not sufficient to maintain the zoonotic agent [9]–[11].

To determine the degree distribution of the bipartite network we analyzed field data on the distribution of the number of ticks (nymphs and larvae) found on hosts (mice and lizards), represented in the model by the degree distribution of host nodes. Exhaustive empirical surveys have shown that macroparasites, and in particular ticks, are aggregated across host populations. This aggregative behavior has important implications for the population and evolutionary dynamics of the parasite and its host [12]–[14].

**Figure 2:**
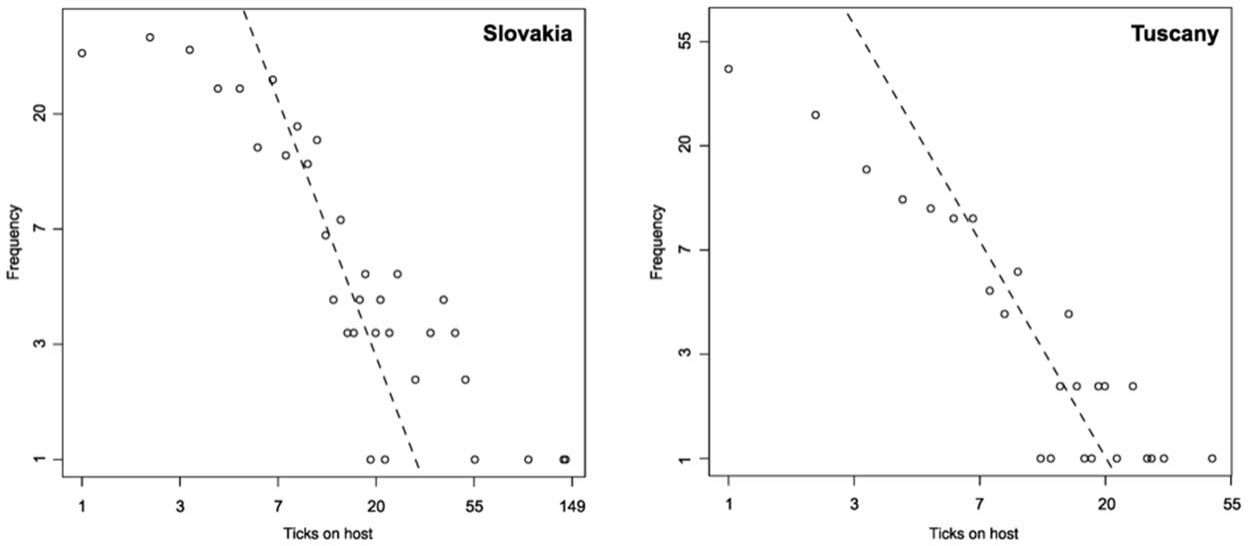
Distribution of the number of ticks on hosts for the Slovakia and Tuscany datasets. The line represents the power-law distribution with parameters 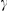 and 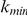 determined by Maximum Likelihood Estimation.

We used the methods described in [15] to assess whether the distribution of ticks on hosts is indeed described by a power-law. We conducted the analysis separately on the data reported in Ref. [10] and on our own field data [16]. As explained in [15] when fitting an empirical distribution to a power law not only the 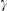 exponent, but also the cutoff 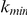 above which the data follow the power-law must be determined. Table 1 shows that a power-law distribution with exponent 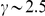 describes the *right tail* of the distribution better than the commonly used negative binomial distribution in both empirical datasets. The power-law distribution is compatible with both datasets (respectively 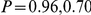 from the goodness of fit test), while the negative binomial can be excluded (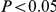). The empirical data and the best fitting power-law distributions are shown in Fig. 2.

**Table 1:** Distribution of vectors on hosts.

Dataset	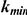	N	Power law		Neg. bin.		LR test	
			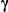	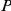	size	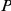	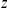	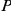
Slovakia [26]	9	116	2.59	0.96	0.81	0.01	3.48	5.0e-4
Tuscany [16]	6	68	2.51	0.70	0.28	0.04	1.70	0.08

The right tail of the distribution of vectors (larvae and nymphae) on hosts (mice and lizards) is described by a power-law distribution. For each dataset we report: the MLE estimate of the value 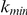 of 
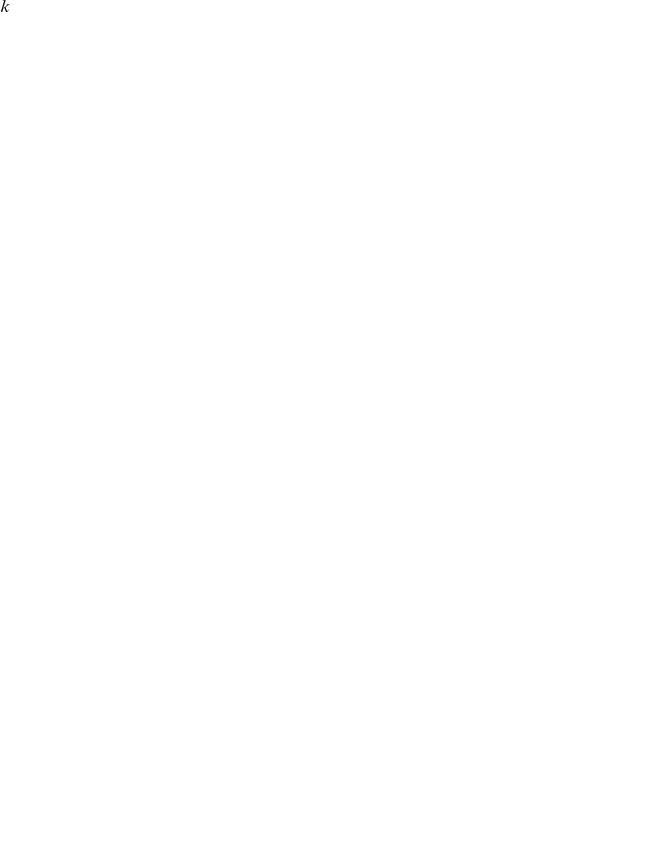
 above which the data are well described by a power-law; the MLE estimate 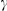 of the power-law exponent; the P-value of the goodness-of-fit test for the power-law; the MLE estimate of the size of the negative binomial distribution; the P-value of the goodness-of-fit test for the negative binomial; the 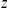-statistic and the P-value of the Vuong test comparing the two distributions.

**Figure 3:**
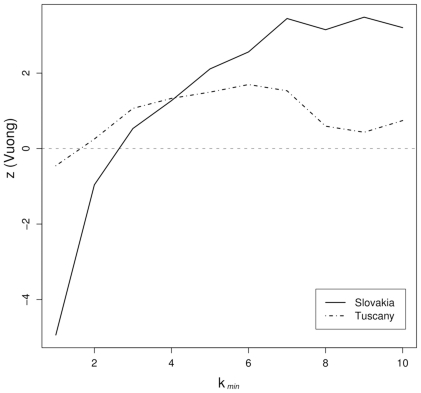
The dependence of the Vuong 
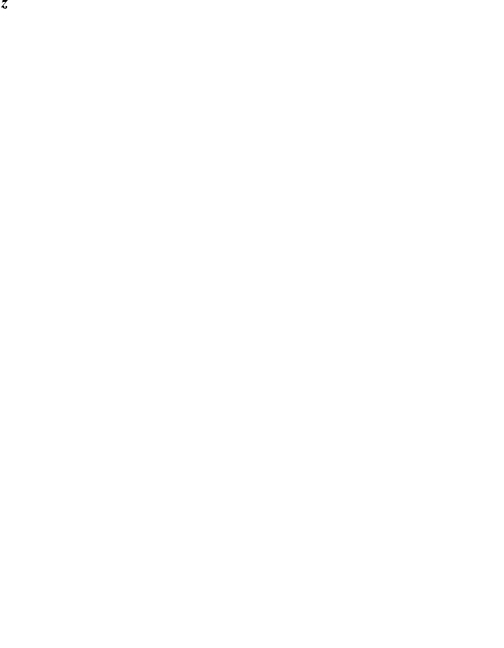
-statistic on the degree threshold 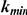 for the Slovakia and Tuscany datasets. Positive (negative) values of 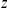 imply that the data are better described by the power law (negative binomial) distribution. While the whole distribution is better described by the negative binomial, the power law is a better fit to the large-
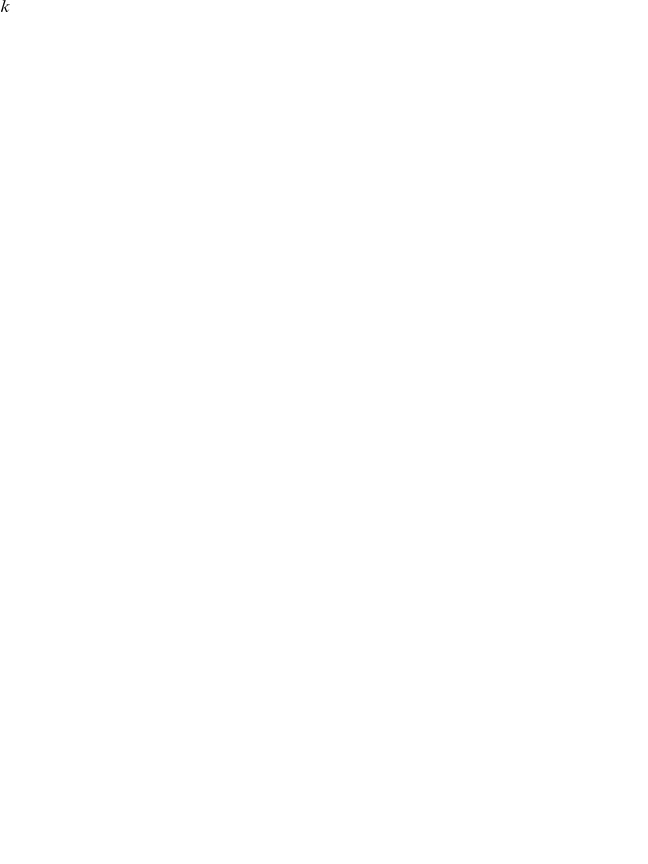
 behavior, which governs the asymptotic behavior of the epidemic threshold.

Let us stress that this applies to the right tail of the distribution: it is instructive to plot the log likelihood ratio of the comparison between power-law and negative binomial as a function of 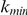 (Fig. 3): the negative binomial does fit the data better than a power-law for low values of 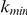, but the tail of the distribution is better described by the power law. This might explain why the negative binomial is commonly used to model the distribution of vectors on hosts. On the other hand, only the right tail of the distribution is relevant to the asymptotic behavior of the epidemic threshold.

**Figure 4:**
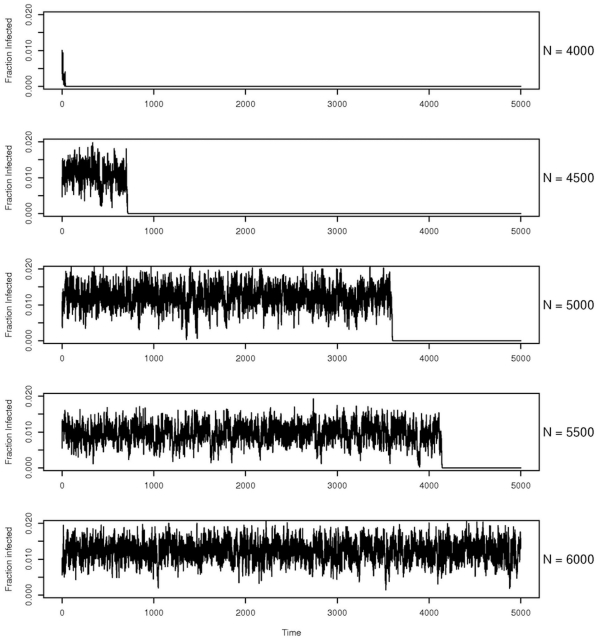
Dynamics of the fraction of infected nodes at constant transmission probability. The networks follow a scale-free degree distribution with 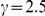 for the hosts and a Poisson degree distribution for the vectors. As the number 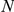 of hosts is increased, the epidemic is sustained for longer times.

Therefore we predict that in this system the epidemic threshold will decrease as the network size increases and will vanish in the limit of infinite network size. Figure 4 shows the time-dependence of the fraction of infected nodes in networks of increasing size at the same value of the transmission probability: larger networks are able to sustain the epidemic for longer times; once the network size crosses a critical value, the epidemic is sustained indefinitely.

## Discussion

The distribution of vectors on hosts plays a fundamental role in the transmission of vector-borne diseases. Since the work of Woolhouse and coauthors [17], the aggregative distribution of ectoparasites on hosts was recognized as a key factor in the transmission of pathogens: “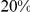 of host population contributes at least 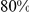 of transmission potential”. This aggregative behavior explains field observations such as those by Perkins and coauthors [18], who observed that the removal of the most-infested mice could reduce the potential transmission by 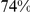 to 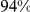.

The negative binomial distribution was proposed in 1998 [19] to describe the aggregative nature of the distribution of macroparasites on hosts. Most mathematical models for tick-borne diseases are based on this assumption. This law faithfully describes the leftmost part of the distribution of ticks on hosts. However our analysis shows that the negative binomial fails to describe the right tail of the distribution of ticks on their hosts, which is instead well described by a power-law decay. This implies that the epidemic threshold decreases with the size of the network and vanishes asymptotically. These results are in agreement with the observation [20] that the density of rodent hosts (and the abundance of their food source) is a much stronger predictor of the density of infected nymphs compared to non-network related factors such as weather and density of deers.

Our results were obtained using a bipartite network to model the spread of the disease. Deviations from strict bipartitedness, provided for example by transovarial transmission, do not alter the main conclusion of an asymptotically vanishing epidemic threshold. Indeed the addition of edges to a network can only decrease the epidemic threshold. Therefore if the bipartite network obtained by considering only edges between vectors and hosts is such that the epidemic threshold vanishes asymptotically, all networks obtained from it by adding non-bipartite edges will have the same property.

In conclusion, we have shown that the extreme aggregation of vectors on hosts, described by the power-law decay of the degree distribution, has dramatic consequences on the behavior of the epidemic threshold, and must therefore be taken into account when modeling the spread of those vector-borne diseases that can be described as propagating on a bipartite network.

## Materials and Methods

### Simulations

#### Network generation

The networks used in the simulations had the same number of vectors and hosts, ranging from 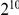 to 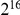. To generate the networks we first assigned “edge stubs” to the nodes of the two classes according to their respective degree distributions and them randomly joined the stubs to produce the network.

We used a pure power-law distribution as a scale-free distribution
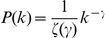
where
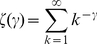
(3)and 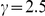; and the Poisson distribution
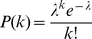
(4)for random degree distributions, with 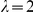.

#### Simulations

We produced 10 independent realizations of each network. For each realization we simulated the SIS model with recovery probability equal to one and initial fraction of infected nodes equal to 1/2. The system was allowed to thermalize for a number of iteration equal to the number of nodes; then the mean and standard error of the number of infected individuals was computed in successive windows of 1,000 iterations each. We considered the system thermalized when the mean of a window was within one standard error from the mean of the previous one.

By varying the transmission probability we located the epidemic threshold for each network realization with a resolution of 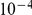 in the transmission probability (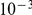 in the random-random case). These 10 values were then used to compute the mean value of the epidemic threshold for the network. The error on the epidemic threshold is taken as the greater value between the standard error computed from the 10 realizations and the resolution in transmission probability.

The dynamics simulations shown in Fig. 4 were performed on networks where the degree distribution is scale-free with 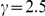 for the hosts and Poissonian for the vectors.

#### Theoretical predictions

The theoretical predictions for the size-dependence of the epidemic threshold shown in Fig. 1 are based on Eq.1 applied to the appropriate degree distributions. Two technical issues arise in the computation:

1. The finite size behavior of 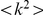 in the scale-free degree distribution is driven by the existence of a cutoff value 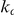 due to the finite size of the network [21]. 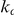 can be estimated [22] by requiring
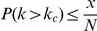
where 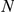 is the number of nodes and 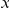 is the number of nodes with 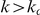 expected in the network: 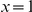 is used in [22], while we found that better agreement with simulation data can be found using a smaller 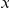. The data in Fig. 1 are obtained with 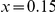. To evaluate the theoretical epidemic threshold we cut the degree distributions at 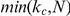.
2. For Poisson degree distributions, Eq. (4), 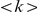 and 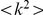 in Eq. (1) must be computed only over nodes with 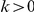, giving:
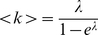
(5)

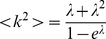
(6)

### Field data analysis

The “Tuscany” dataset contains infestation data from field work conducted in a natural reserve in Le Cerbaie hills, Pisa province, central Italy. This area is characterized by Mediterranean vegetation, an optimal habitat for ticks and their hosts [23], and previous studies reported the presence of *B. burgdoferi* [24], [25]. The sampling sessions were carried out, monthly, from March to August 2006. Rodents and lizards were captured as described in [25]. Two different hosts were considered, *Apodemus* spp. and *Podarcis* spp. The animals were infested only by immature ticks *Ixodes ricinus*. The number of mice and lizards examined are reported in Table 2.

**Table 2:** Field data.

	mice	lizards	total
collected	161	86	247
non-infested	51	13	64
larvae-infested	110	58	168
nymphs-infested	19	44	63
co-infested	19	29	48

Number of mice and lizards examined to determine the distribution of vectors on hosts in the Tuscany dataset.

The “Slovakia” dataset refers to the distribution of larvae and nymphs on mice, obtained from Fig. 3 of Ref. [26]. These data were collected in the area of Bratislava (Slovakia), where TBE and Lyme disease are present.

The power-law
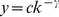
(7)is assumed to describe the right tail of the degree distribution, that is the nodes with degree 
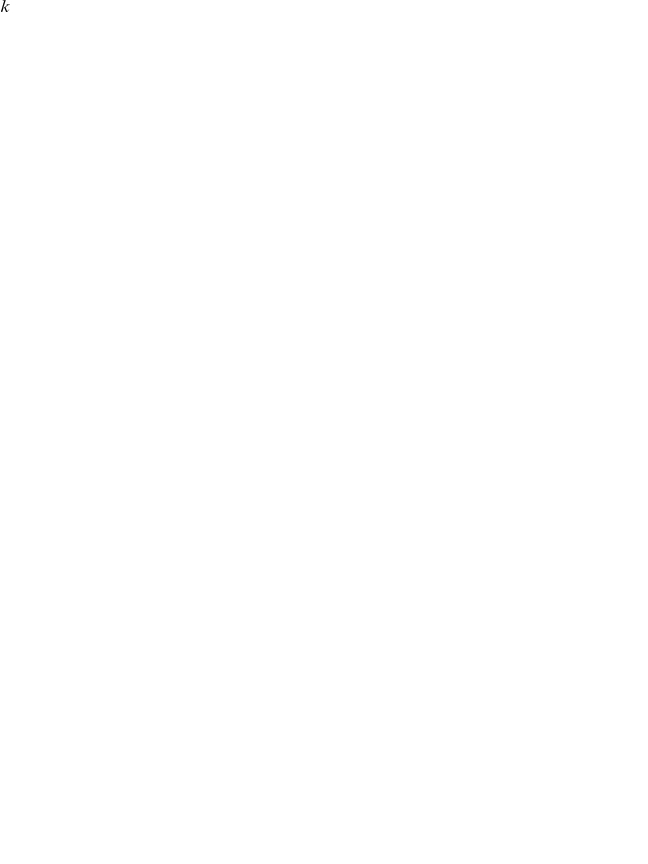
 greater then or equal to a minimum 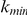. 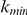 was obtained with the method introduced in [15], [27] and based on the Kolmogorov-Smirnov (KS) statistic. 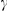 was estimated by maximum likelihood estimation (MLE) performed on the data with 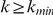. MLE was also used to find the parameters of the negative binomial distribution
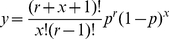
(8)providing the best fit to the same data.

The goodness-of-fit tests were based on the empirical distributions of the Kolmogorov-Smirnov statistic obtained by Monte Carlo methods as explained in [15]. Power-law and negative binomial distributions were compared using the Vuong test on likelihood ratios.
